# Clinical Patterns of Cerebral Palsy in Pediatric Patients From Tertiary Referral Hospitals in Dubai, United Arab Emirates: A Retrospective Observational Study From 2018 to 2020

**DOI:** 10.7759/cureus.106850

**Published:** 2026-04-11

**Authors:** Meera Almheiri, Alya Al Ameri, Hadi A Helali, Abdulla Alawadhi, Samar Almuntaser

**Affiliations:** 1 Medicine, Mohammed Bin Rashid University of Medicine and Health Sciences, Dubai, ARE; 2 Pediatric Neurology, Al Jalila Children's Hospital, Dubai, ARE

**Keywords:** cerebral palsy (cp), clinical manifestations, management, pediatric population, perinatal asphyxia, spastic cerebral palsy, spasticity disorder, tertiary referral hospital

## Abstract

Background

Cerebral palsy (CP) is a neurological condition characterized by a combined impairment of posture, movement, and motor function, as well as potential sensory, neurological, and musculoskeletal problems. It is a chronic disorder caused by non-progressive aberrations in the evolving fetal or newborn brain.

Objectives

To examine the potential association between different comorbidities, genders, and clinical patterns associated with CP and to observe the prevalence of CP in Dubai, United Arab Emirates (UAE).

Methods

This was a retrospective observational study using previously collected data from 283 pediatric patients aged 18 years or younger diagnosed with CP at the Al Jalila Children's Hospital and Latifa Women and Children’s Hospital. Data collection period spanned from January 1, 2018, to December 31, 2020. Data was analyzed using chi-square and proportion tests.

Results

Two hundred and thirty-one patients (89.9%) presented with spastic CP. Quadriplegia was the most prevalent type of spastic topography, which was found in 103 patients (44.6%), followed by diplegia (85 patients, 36.8%). Perinatal asphyxia was the most common etiology (150 patients, 52.7%). There was no statistically significant correlation between demographic data and the different comorbidities.

Conclusions

The study showed that spastic quadriplegia was the most common type of CP in this Dubai cohort. Perinatal asphyxia was the most common etiology.

## Introduction

Cerebral palsy (CP) is a neurological disorder characterized by impairments of posture, movement, and motor function, along with potential sensory, neurological, and musculoskeletal problems. It is a long-term disease caused by non-progressive damage in the evolving brain [1].

Children with CP often have poor muscle tone, difficulty standing, and irregular posture [2]. Common comorbidities include seizures/epilepsy and developmental delay [3]. CP is not a progressive disease, but its symptoms may vary over time [4].

There are five main types of CP based on the main movement disorder seen and the location of the insult. The first and the most common is spastic, characterized by increased muscle tone resulting from cortical damage; dyskinetic, involving involuntary movements due to basal ganglia injury; ataxic, marked by impaired balance and coordination associated with cerebellar dysfunction; and mixed, in which a child presents with features of more than one type [5]. In addition, the topographic map usually follows the type of CP. Diplegia is when half of the four limbs, with legs being more affected than arms, hemiplegia, which is when only one side of the child’s body is affected, and lastly quadriplegia, in which all the four limbs, including the face and trunk, are affected [6].

The causes of CP are numerous, complex, and depend on many factors, the most common being birth asphyxia, which is oxygen deprivation to the placenta leading to hypoxia. It can occur due to multiple reasons. If hypoxia lasts long, it can cause permanent damage to the brain, leading to the neurodevelopmental abnormalities seen in cerebral palsy [7]. The variation and inaccurate diagnosis of birth asphyxia have led to a wide range of prognoses for birth asphyxia. According to a prior study, the percentage of CP patients with birth asphyxia ranged from 3% to over 50% [8]. Other less common causes may include genetic factors, brain structural defects, physical brain insults, etc. [9].

Data regarding CP in the United Arab Emirates (UAE) is limited. To the authors' best knowledge, this is the first study in the UAE to examine the clinical patterns, etiologies, and disabilities associated with CP. This study sought to identify the aforementioned in patients from the Al Jalila Children’s Hospital and Latifa Hospital in Dubai, UAE, as well as assess if there is an association between demographics and some of the main comorbidities of CP (Global Developmental Delay (GDD), and epilepsy).

## Materials and methods

Study design and setting

This was a retrospective observational study conducted in the pediatric neurology department at the Al Jalila Children’s Hospital and Latifa Children’s Hospital, the two main neonatal and pediatric hospitals in Dubai. These hospitals serve both Emirati citizens and expatriate families. The study period spanned from January 1, 2018, to December 31, 2020. It included all pediatric patients aged one month to 18 years diagnosed with CP. The data were collected using the Epic Electronic Health Record (EHR) database (Salama, Epic Systems Corporation, Wisconsin, USA). The Institutional Review Board (IRB) of the Mohammed Bin Rashid University of Medicine and Health Sciences approved the study (approval no. MBRU IRB-2022-113). Informed consent from the patients was waived by the MBRU IRB, given that the study is a retrospective chart review, hence carries minimal to no risk, and can be carried out with the waiver, and because all the patients admitted to the hospital signed a consent on admission that their chart data will be used in future research.

Participants and eligibility criteria

All patients aged one month to 18 years who were seen in outpatient clinics and diagnosed with CP during the study period were eligible. The CP diagnosis was based on the physicians’ clinical assessment documented in the electronic record. Exclusion criteria were: 1) age younger than one month (neonates) or older than 18 years (adults); 2) incomplete birth-related data; 3) other classes of neurodegenerative disorders. These criteria were chosen to create a homogenous cohort and minimize confounding.

Selection bias was present in this study, as most participants were Emirati, limiting generalizability to the Emirati population. However, selection bias was minimized by using databases from two hospitals in Dubai, which made it more generalizable to Emiratis in Dubai and, to an extent, in the UAE, but not as generalizable to the region or the world. Information bias could be present because this research used secondary data, which may lead to missing data. Additionally, since the database was maintained by different doctors, there may be bias in the data collection.

Sample size

Based on the inclusion and exclusion criteria, 283 patients from the Latifa Children’s Hospital and Al Jalila Children’s Hospital were identified for inclusion in this study. This larger sample size improved the reliability of the findings and allowed for more detailed analysis across CP subtypes.

Data sources and variables

Electronic medical records were screened using Epic (locally referred to as 'Salama'). Lists of clinic visits were filtered to identify infants with a clinical diagnosis of CP. Patient charts were then reviewed to confirm eligibility. Data was abstracted into a password‑protected spreadsheet. Data collection involved a detailed review of existing datasets and medical records available in the Salama database, with a focus on pediatric patients diagnosed with CP. The data extracted included demographic information, clinical characteristics, and comorbidities associated with CP. Key variables recorded were comorbidities (GDD and epilepsy). 

The primary data sources were electronic medical records and standardized assessment forms used in clinical practice at the hospital. Outpatient clinic records were thoroughly reviewed to document comorbidities and demographic information, including patients' age, gender, nationality, type of CP, associated topography, and whether the patient had the following comorbidities: GDD and epilepsy. The data were collected to determine the most prevalent value for each variable and to identify a relationship between comorbidities and gender. Etiologies such as hypoxia, stroke, hypoxic-ischemic encephalopathy (HIE), and agenesis of the corpus callosum were grouped under perinatal asphyxia. Brain structure included conditions such as hemiatrophy and kernicterus. Brain injuries included shaken baby syndrome and road traffic accidents (RTA) accidents that caused brain injuries. The variable “other” included different types of metabolic disorders that affect the brain. Finally, patients were divided based on their age groups: infants (ages 0-2), children (ages 3-11), and adolescents (ages 12-18).

Statistical analysis

Data was analyzed using IBM SPSS Statistics for Windows, Version 25 (Released 2017; IBM Corp., Armonk, New York, United States). The baseline data were assessed for normality to determine the appropriate statistical measures. Continuous variables, including age in months, age at presentation, birth weight, and neonatal intensive care unit (NICU) days, were summarized using means and standard deviations (SD). Categorical variables, such as gender, nationality, and the presence of specific comorbidities (e.g., epilepsy, visual impairment), were summarized as counts and valid percentages.

Data were stratified by CP type into subgroups. The proportions of children with CP presenting with each subgroup were calculated and presented as percentages, allowing comparisons across the different types of CP. Descriptive statistics were employed to characterize the study population and summarize the prevalence of comorbidities, while inferential statistical method (chi-square test), was used to explore associations between demographic factors and clinical outcomes. A two‑sided p<0.05 was considered statistically significant.

## Results

Participants

A total of 283 pediatric patients below the age of 18 were diagnosed with CP in the Al Jalila Children’s Hospital and Latifa Children’s Hospital between January 1, 2018, and December 31, 2020. Most patients (n=63, 92.9%) were aged between three and 11 years.

Patient characteristics

Most of the cohort was made up of male patients (n=161, 56.9%). The most common type of CP in the collected data was spastic CP, with 231 patients (89.9%). Spastic CP was further divided into paralysis topography as follows: quadriplegia (n=103, 44.6%), followed by diplegia (n=85, 36.8%).

Regarding the etiology of CP, perinatal asphyxia was most common (n=150, 52.7%). Although it was the most common cause, there was no statistically significant correlation between its prevelance and demographic factors.

It was observed that GDD was the most common comorbidity associated with CP (n=215, 84.6%). Additionally, another common comorbidity was seizures, defined as one episode of an epileptic seizure (present in n=146 patients, 57.5%), followed by epilepsy, defined as two or more epileptic seizures (present in n=130, 51.2%) (Table 1).

**Table 1:** The variables and the clinical patterns observed in this study

Variables	Number of patients (percentage) out of the total (n=283)
Gender	
Male patients	161 (56.9%)
Female patients	122 (43.1%)
Age categories	
0-2 years old	18 (6.4%)
3-11 years old	263 (92.9%)
12-18 years old	2 (0.7%)
Types of cerebral palsy (CP)	
Spastic CP	231 (89.9%)
Mixed CP	13 (5.1%)
Hypotonic CP	7 (2.7%)
Dyskinetic CP	3 (1.1%)
Ataxic CP	3 (1.1%)
Spastic topography	
Quadriplegia	103 (44.6%)
Diplegia	85 (36.8%)
Hemiplegia	27 (11.7%)
Triplegia	12 (5.2%)
Others (double hemiplegia, monoplegia)	4 (1.7%)
Etiology	
Perinatal asphyxia	150 (52.7%)
Genetic	27 (9.5%)
Brain structural defect	11 (2.8%)
Brain insult	9 (2.8%)
Other	5 (1.85%)
Unknown etiology	86 (30.4%)
Presented with global development delay (GDD)	
Yes	215 (84.6%)
No	39 (15.4%)
Presented with epilepsy	
Yes	130 (51.2%)
No	124 (48.8%)
Presented with seizure	
Yes	146 (57.5%)
No	108 (42.5%)

**Figure 1:**
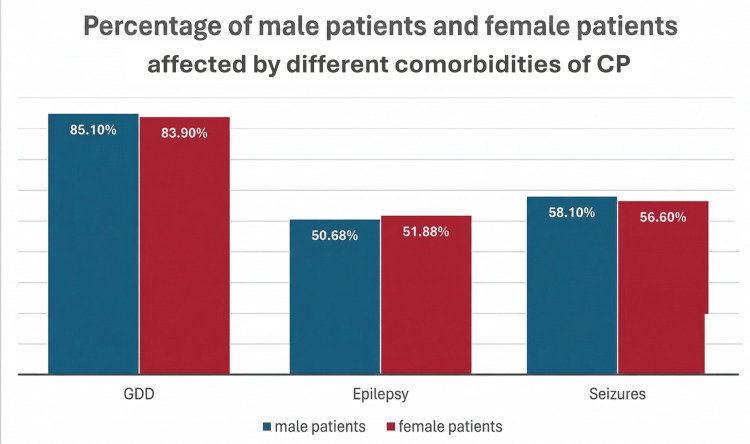
Results of the cross-tabulation of different comorbidities and gender CP: Cerebral palsy; GDD: Global Developmental Delay.

To further investigate, a cross-tabulation was conducted to find an association between gender and the three comorbidities This was done in 254 patients. GDD was seen in 89 female patients and 126 male patients. This was not statistically significant (p-value=0.789). Epilepsy was seen in 55 female patients and 75 male patients, with a p-value of 0.849, showing no significance. Finally, 60 female patients and 86 male patients presented with seizures, also showing no significance with a p-value of 0.811. The results concluded that there was no association between gender and the studied comorbidities (Figure 1 and Table 2).

**Table 2:** Association between the gender and prevalence of comorbidities in patients with cerebral palsy GDD: Global Developmental Delay.

Comorbidity	Male (n=148)	Female (n=106)	Total (n=254)	p-value
GDD	126 (85.10%)	89 (83.90%)	215 (84.60%)	0.789
Seizures	86 (58.11%)	60 (56.60%)	146 (57.50%)	0.811
Epilepsy	75 (50.68%)	55 (51.89%)	130 (51.20%)	0.849

## Discussion

Summary of major findings

Among our patients, common findings and observations emerged. Most patients were children aged 3-11 years, with a minority of infants and adolescents. The most common type of CP was spastic (stiff muscles), and the specific topography was quadriplegia (all four limbs affected, including the face and trunk) [4]. Regarding etiologies, perinatal asphyxia had the highest number of patients, while others, like genetic, brain structural damage, and brain insults, were found in smaller frequencies. GDD is the highest comorbidity observed. Male patients had more comorbidities than female patients, but the difference was not statistically significant.

Comparison with previous studies

This study was the first to investigate the types of CP and the types of spastic topography in the UAE. Hence, there were no studies to compare with, other than global literature. Nonetheless, a previous study of CP patients from India, showed data that could be reflected in this research. The study reported that the most common type of CP was spastic (65%) and the most common type of spastic topography was quadriplegia (69%) [10].

A similar cross-sectional observational study by Kana et al. (2022) [11] showed that the most frequent etiology was perinatal asphyxia (54.6%). This was also reflected in our study, which showed that perinatal asphyxia was the most common etiology (52.7%). Kana et al. also showed that most patients (95.5%) had intellectual impairment; the present study also concurred with that and proved that most participants had GDD (84.6%). Other than intellectual impairment, the study [11] showed that 69.3% of patients had epilepsy, while our study showed that 51.2% of patients presented with epilepsy. There were no results regarding seizures alone in Kana et al. [11].

Another study by Raja & Hapani (2019) [12] showed that cognitive impairment was present in 77% of patients. Results regarding cognitive impairment were very similar to those found in the present study. Prevalence of epilepsy in patients was 38%. The prevalence of seizures in the current study (57.5%) aligned closely with the 56.2% reported by Raja and Hapani. The study [12] also showed that the most common type of CP was spastic CP (72.5%), similar to the present study.

Strengths and limitations

This was the first study in the UAE observing clinical patterns and etiologies associated with CP. Two large hospital datasets were used, thereby increasing its generalizability.

The secondary data collected primarily included participants from the UAE, limiting generalizability to the Dubai population. In addition, majority of the participants (92.9%) were in the age group of 3-11 years, limiting the variety in exploring the age group of children below three years and children between the ages of 12 and 18 years, and making it less generalizable to the participants above the age of 11 years and below the age of three years [13].

Regarding data, some patients were born outside the UAE, so etiology data were missing. In addition, information was missing regarding other variables, such as the type of CP and comorbidities. This limited the measurement of etiology prevalence and yielded a less precise percentage.

Areas for future research

Future studies should aim to further elucidate the specific etiological factors underlying CP. While our study highlights perinatal asphyxia as a significant contributor, the secondary nature of our data prevented an analysis of specific triggering events, such as placental abruption, uterine rupture, cord prolapse, or hemorrhage [14]. Investigating these granular details would provide a more precise understanding of neonatal risk. Additionally, further research is warranted into the correlation between consanguinity and CP prevalence to better understand the role of genetic factors. Finally, future work could explore the relationship between head circumference - specifically microcephaly, macrocephaly, and hydrocephalus - and long-term neurodevelopmental outcomes in patients with CP [15]. 

## Conclusions

Observing and identifying clinical patterns associated with CP and the likelihood of comorbidities helps raise awareness of the symptoms associated with CP, aids physicians in earlier diagnosis, and provides earlier treatment planning, which could improve the prognosis of the disease and the quality of life of patients. In addition, identifying etiologies associated with CP could reduce risk and help in prevention efforts if it is an acquired condition, such as cases with brain lesions or in preventable cases, such as some cases of perinatal asphyxia.

Furthermore, a comprehensive understanding of the diverse manifestations of CP allows for the development of highly personalized intervention strategies that address both physical and cognitive needs. By shifting the focus to proactive monitoring and multidisciplinary care, healthcare providers can reduce the severity of long-term complications and enhance the individual's independence. Ultimately, these integrated efforts in clinical observation and etiologic research serve as a foundation for advancing global health standards and improving outcomes for those living with CP.
